# Cultural stressors and behavioral correlations of post-pandemic anxiety among Emirati university students

**DOI:** 10.3389/fpsyg.2025.1666403

**Published:** 2025-10-14

**Authors:** Heather Webb, Ayesha Abdulla, Roberta Fenech, Heyam F. Dalky, Yasser Mahmmod, Joelle Chamoun

**Affiliations:** ^1^Higher Colleges of Technology, Al Nahda Campus, Dubai, United Arab Emirates; ^2^RN Faculty of Nursing/WHO Collaborating Center, Jordan University of Science & Technology, Irbid, Jordan; ^3^Animal Medicine and Welfare, Long Island University, Brookville, NY, United States; ^4^Higher Colleges of Technology, University City Campus, Sharjah, United Arab Emirates

**Keywords:** anxiety, university students, the UAE, GAD-7, behavioral coping, Emirati, culturalstigma

## Abstract

This study investigates the prevalence and predictors of anxiety among Emirati university students in the post-pandemic period, focusing on the influence of socio-demographic variables, mental health comorbidities, and lifestyle behaviors. A cross-sectional online survey was conducted across 16 UAE public university campuses from November 2022 to February 2023, yielding 7,244 complete responses from students aged 18 and above. The survey assessed anxiety using the Generalized Anxiety Disorder 7-item (GAD-7) scale and included items on demographics, physical and mental health, support systems, and behavior patterns such as substance use and internet engagement. Multivariable logistic regression models examined associations between anxiety and key explanatory variables. Results revealed that 8% of respondents experienced severe anxiety, with significant associations observed between anxiety and gender, age, income, disability status, and physical health conditions. Females, younger individuals, those with household incomes below AED 10,000, and students with chronic illnesses or disabilities were at higher risk. Mental health comorbidities such as depression, panic attacks, eating disorders, OCD, and ADHD showed strong associations with anxiety severity. Furthermore, behavioral correlations including excessive internet use, gaming, alcohol consumption, and prescribed medication use were linked to elevated anxiety levels, although not all were statistically significant. Despite efforts to increase mental health awareness in the UAE, stigma and cultural norms remain barriers to help-seeking. The study underscores the need for culturally tailored mental health interventions that address stigma, promote early screening, and consider the role of maladaptive coping behaviors. Universities should implement accessible support systems that incorporate Arabic-language resources, peer mentoring, and family or religious engagement. These findings contribute to the global understanding of anxiety in higher education and highlight the importance of localized mental health strategies in post-pandemic recovery.

## 1 Introduction

The COVID-19 pandemic has had extensive impacts on mental health, particularly among university students who encountered distinctive academic, financial, and social stressors. The feelings of uncertainty, isolation, and grief, combined with disrupted routines and evolving academic expectations, have significantly contributed to increased levels of anxiety (Wang X. et al., 2020; Zhao et al., 2021). Although anxiety is a typical stress response, the pandemic has engendered an environment in which coping mechanisms were frequently inadequate or ineffective. Numerous studies have documented a rise in the prevalence of anxiety during this period; however, fewer investigations have examined how cultural and behavioral factors influence these responses within specific populations (Alteneiji, 2023; Bhui et al., 2007; Gouzman et al., 2022; La Rosa and Commodari, 2023; Lai et al., 2020; Losada-Baltar et al., 2020; Malik et al., 2023; Mehareen et al., 2021; Nikolaev, 2021; Son et al., 2020; Soria et al., 2021; Xiong et al., 2020).

In the United Arab Emirates (UAE), cultural expectations concerning academic achievement, familial obligations, and the stigma surrounding mental health significantly influence students’ experiences and responses to stress. Traditional values, including honor, interdependence, and family solidarity, may deter individuals from seeking help, whereas modernization, urbanization, and increased exposure to global influences foster autonomy and self-expression (Alteneiji, 2023; Bhui et al., 2007). These factors contribute to a culturally intricate environment in which anxiety manifests through both conventional and unconventional means. The prevalence of mental health stigma across Arab cultures further complicates access to care (Jones-Lavallée and Leanza, 2025). While initiatives aimed at enhancing health behaviors and increasing mental health awareness are underway, there remains a notable lack of studies that explore how students perceive and engage with these behaviors as coping mechanisms.

Health behaviors such as overeating, substance use, and excessive engagement with the internet are frequently employed, either consciously or unconsciously, as mechanisms for regulating stress and emotional discomfort (DeSteno et al., 2013; Kaplan et al., 2024; Rahman et al., 2025; Sheeran et al., 2013). However, individuals often lack awareness of the relationship between their emotional states and these behaviors, particularly when the responses are implicit rather than deliberate (Kruglanski et al., 2018; Yang et al., 2024). This absence of insight poses a challenge to conventional definitions of coping and suggests that anxiety may lead to maladaptive behavioral patterns with long-term implications. Within academic contexts, particularly among students in high-stress environments, these behaviors may become normalized and remain unaddressed (Amanvermez et al., 2024; Fink, 2016).

This study aims to investigate anxiety among university students in the United Arab Emirates during the post-pandemic period, with particular emphasis on gender norms, stigma, health-related coping behaviors, and comorbid mental health conditions. While prior research has documented mental health challenges experienced during the pandemic, the ongoing ramifications of these disruptions continue to emerge (Lin et al., 2024; Otaki et al., 2025; Vajpeyi Misra et al., 2022). By examining the sustained psychological impact of the pandemic within the unique socio-cultural context of Emirati students, this study contributes to the formulation of culturally sensitive interventions aimed at enhancing student well-being in higher education (Badrasawi and Zidan, 2019; González-Alonso et al., 2019; Keller et al., 2008; Theurel and Witt, 2022).

## 2 Methods

### 2.1 Participants and recruitment

A cross-sectional online survey was conducted from November 2022 to February 2023 across 16 campuses of one public higher education institution in the UAE. The study specifically targeted registered Emirati students aged 18 years and older. As of the 2022–2023 academic year, the institution had reported a total of 42,614 registered students (Ministry of Education, 2022). The sample size was not determined by a minimum calculation, but was instead strategic, aiming to maximize the response rate from the entire target population of 42,614 Emirati students across all 16 campuses of one public institution. Utilizing official university communication channels allowed for a widespread distribution of the survey. The final sample of 7,244 participants represents a response rate of approximately 17%, which is considered robust for a broad email-based survey and provides exceptionally high statistical power to detect even small effect sizes in the data.

The central services administration of the university oversaw the recruitment and data collection processes. A standardized email was sent to all registered Emirati students. This email included a link to the SurveyMonkey platform and clearly presented the purpose and objectives of the study, specifically designed to enhance the understanding of issues pertaining to the physical and mental health, as well as the overall well-being, of students. It was expressly stated that participation in the study was voluntary, anonymous, and confidential. The email provided a comprehensive overview of the informed consent procedure, which was incorporated into the first page of the online survey; participants were required to digitally acknowledge their consent before advancing to the questionnaire items. The study received ethical approval from the university’s institutional review board (HCT Fund 213783).

The survey instrument was professionally translated and culturally adapted to align with UAE norms and was available in English, the official language of instruction at the participating institutions, where proficiency in both English and Arabic is a requirement for enrollment. The study was exploratory in nature, with the objective of mapping the landscape of physical and mental health, and well-being in a post-pandemic context, while also identifying relevant cultural correlations. We expected to achieve a comprehensive dataset that would allow us to (1) quantify the prevalence of mental health symptoms, (2) identify key cultural stressors significantly associated with these symptoms, and (3) develop a nuanced model of the relationships between cultural factors and student well-being, thereby informing future interventions aimed at providing support.

### 2.2 Survey design

The survey consisted of four main sections: demographics, physical health, mental health, and support. The demographic section included questions on gender, nationality, age range, university and location, program and year of study, employment status, marital status, children, household income, and whether participants were registered with the UAE Ministry of Health as having a disability. The physical health section addressed participants’ general health, exercise habits, and sleep patterns. The mental health section covered diagnoses of mental or developmental disorders, stress levels, burnout, exposure to negative events, symptoms of anxiety, and additional factors such as loneliness, self-harm, bullying, attitudes toward mental health, the impact of COVID-19, and behavioral or substance addictions. The final section on support assessed perceptions of academic support, the learning environment, progress toward degree completion, and sense of community.

Anxiety was assessed using the Generalized Anxiety Disorder 7-item (GAD-7) scale, a validated screening tool commonly used in mental health care settings to identify symptoms of anxiety (Spitzer et al., 2006; Wang Y. et al., 2020). The GAD-7 includes seven items that evaluate symptoms such as nervousness, uncontrollable worry, restlessness, irritability, and fear of impending negative events (Delgadillo et al., 2012; Nunes et al., 2022). Participants rated how frequently they experienced each symptom over the past 2 weeks. Scores range from 0 to 27, with severity classified as minimal (0–4), mild (5–9), moderate (10–14), or severe (15–21) (Spitzer et al., 2006). The GAD-7 has demonstrated strong reliability and internal consistency, with Cronbach’s *α* coefficients supporting its use (Liu and Shi, 2023; Mossman et al., 2017). It is significantly correlated with clinical anxiety symptoms and shows moderate associations with depression, making it a robust tool for psychological assessment in both clinical and research contexts (Delgadillo et al., 2012; Nunes et al., 2022).

### 2.3 Data analysis

The first step was to screen the data to identify and address any unlikely or missing values before conducting any statistical analysis (López-Serrano et al., 2021). After that, a descriptive statistical analysis of the potential factors associated with the main variables of interest, such as gender, age, employment, household income, disability status, presence of physical health conditions, and treatment for physical health conditions, was performed. The study examined various additional explanatory variables associated with mental health disorders, such as depression, panic attacks, substance use disorder, eating disorder, bipolar disorder, OCD, ADHD, autism spectrum disorder, dyslexia, experiences of cyberbullying, and daytime sleep patterns. Additionally, the research explored behavior-related variables, including vaping, smoking, illicit drug use, prescribed medication, alcohol consumption, gaming, internet usage, and gambling.

The explanatory variables of interest were classified into three main categories, including socio-demographic characteristics of our study population (11 variables), mental disorders (17 variables), and behavior disorders (8 variables). We run three different multivariable logistic regression models with anxiety expressed as “yes” versus “no” and relevant explanatory variables of each category (López-Serrano et al., 2021). To examine the relationships between the dependent and independent variables, all relevant variables were included in a multivariable logistic regression for each category. Prior to analysis, we confirmed that multicollinearity was not a concern; all variance inflation factors (VIF) values were below 3. A *p*-value of < 0.05 was used to denote statistical significance. A manual backward elimination process was applied, removing variables with a *p*-value greater than 0.05 (Dohoo et al., 2010). The final model retained only variables with a *p*-value below 0.05. For each variable, the *p*-value, odds ratio, and 95% confidence interval (95% CI) were recorded (López-Serrano et al., 2021). All statistically significant results (*p*-value < 0.05) were included in the final model. Statistical analyses were performed using R version 3.3.3 (R Core Team, 2017). For the model diagnostics, all relevant predictors were initially included in the models, and a manual backward elimination procedure was applied, sequentially removing variables with *p* > 0.05. The final models retained only predictors with *p* < 0.05. Model diagnostics were performed, including assessment of multicollinearity using variance inflation factors (VIF), and evaluation of model fit using the Hosmer–Lemeshow goodness-of-fit test and Akaike Information Criterion (AIC). This information has been added to the text.

## 3 Results

### 3.1 Demographics

The analysis included 7,244 (85.5%) complete responses, while 1,198 were excluded due to missing values or incomplete data. The participant demographic comprised 4,559 (62.9%) females and 2,685 (37.1%) males. Among them, 418 (5.8%) were working students, and 6,826 (94.2%) were non-working students. Regarding marital status, 6,812 (94%) participants were single, 384 (5.3%) were married, 36 (0.5%) were divorced, 6 (0.08%) were separated, and six (0.08%) were widowed.

The participant age distribution was highly concentrated, with the vast majority (96%) being between 18 and 24 years old. Given this limited variability and the research focus on the shared cultural context of university life, age was treated primarily as a control variable. The categorization was pragmatically determined to ensure sufficient group sizes for meaningful statistical comparison while still capturing potential life-stage differences. The group of 25–34 was retained to include postgraduate and mature students, while the small number of participants aged 35 + were analyzed as a single group to avoid cell sizes too small for analysis.

In the study program, the distribution based on the year of study is as follows: 564 (7.8%) students in the foundation year, 1,997 (27.6%) in the first year, 2,089 (28.8%) in the second year, 1,605 (22.2%) in the third year, 973 (13.4%) in the fourth year, 13 (0.2%) pursuing a master’s degree, and three (0.04%) pursuing a Ph.D. Regarding employment status, 418 (5.8%) students are employed, while 6,826 (94.2%) are unemployed.

In the context of student demographics regarding parenthood, a total of 236 students, representing 3.3%, have children, whereas 7,008 students, accounting for 97.8%, do not have any children. Household income distribution is as follows: 1,512 (20.9%) students have an income below 10,000 AED; 3,804 (52.5%) have an income between 10,000 and 30,000 AED; 1,332 (18.4%) have an income between 30,000 and 60,000 AED; and 596 (8.2%) have an income above 60,000 AED.

Regarding disability, 177 (0.02%) students are registered with the government as having a disability, while 7,067 (97.6%) are not. Among the students, 730 (10.1%) are diagnosed with a physical health issue, while 6,514 (89.9%) are not. Additionally, 685 (9.5%) students receive treatment for a physical health issue, while 6,559 (90.5%) do not.

### 3.2 Generalized anxiety disorder responses

The overall GAD-7 scale demonstrates a high level of reliability with a Cronbach’s *α* coefficient of 0.93, surpassing the recommended value of 0.80, indicating excellent reliability (Löwe et al., 2008; Spitzer et al., 2006). As indicated in Table 1, the majority of students reported minimal anxiety.

**Table 1:** Generalized anxiety score.

Total Score GAD – 7	Anxiety severity	
0–4	4,119 (58.2%)	Minimal
5–9	1,642 (23.2%)	Mild
10–14	742 (10.5%)	Moderate
15–21	569 (8%)	Severe

### 3.3 Association of anxiety with socio-demographic characteristics

The results of the final model for the association of anxiety with socio-demographic characteristics using a multivariable logistic regression model are listed in Table 2. The final multivariable model revealed that gender, household income, being registered as having a disability, being employed, rate of physical health, being diagnosed with physical health issues, and receiving treatment for physical health issues were significantly (*p* < 0.05) associated with anxiety in the studied population. Age was a borderline of statistical significance, but it was kept in the final model because of the interaction effect with the gender variable. Male participants had 0.67x lower odds of anxiety than female participants (*p* < 0.05). Younger age participants had higher odds of having anxiety compared to older participants. The lower the salary, the higher the odds of suffering from anxiety, where participants with a household income under AED 10,000 have a higher risk of anxiety at 1.33 (95% CI: 0.93–1.78). Participants who were registered as having a disability have a higher risk of anxiety at 2.23 (95% CI: 11.51–3.26). Participants diagnosed with physical health issues have a higher risk of anxiety at 1.47 (95% CI: 1.10–1.94). Participants who are receiving treatment for physical health issues have a higher risk of anxiety at 1.64 (95% CI: 1.23–2.17). The poor rate of physical health had higher odds of having anxiety, 4.47 (95% CI: 2.99–6.66), compared to other rates. The participants who are employed had higher odds of having anxiety, 1.35 (95% CI: 0.97–1.85), compared to non-employed participants.

**Table 2:** Results of the final model for the association of anxiety with socio-demographic characteristics using a multivariable logistic regression model.

Item	Level	OR	95% CI	*p* value
Intercept	---	0.058	0.04–0.084	<0.0001
Gender	Female	Ref	Ref	0.0009
	Male	0.67	0.55–0.85	
Age	< 24	Ref	Ref	0.070
	< 34	1.61	1.099–2.315	
	< 44	1.40	0.451–3.557	
	45	1.74	NA–1.673	
Salary	Above AED 60,000	Ref	Ref	0.0008
	Between AED 10,000 and 30,000	0.88	0.65–1.20	
	Between AED 30,000 and 60,000	0.81	0.57–1.15	
	Under AED 10,000	1.3	0.93–1.78	
Employed	No	Ref	Ref	0.044
	Yes	1.35	0.97–1.85	
Rate of physical health	Excellent	Ref	Ref	<0.0001
	Poor	4.47	2.99–6.66	
	Fair	2.25	1.66–3.07	
	Good	1.74	1.32–2.33	
	Very good	1.19	0.88–1.63	
Registered as having a disability	No	Ref	Ref	<0.001
	Yes	2.23	1.51–3.26	
Diagnosed with physical health issues	No	Ref	Ref	<0.0001
	Yes	1.47	1.10–1.94	
Receiving treatment for physical health issues	No	Ref	Ref	<0.001
	Yes	1.64	1.23–2.17	

### 3.4 Association of anxiety with mental health disorders

The results of the final model for the association of anxiety with mental health disorders using a multivariable logistic regression model are listed in Table 3. The final multivariable model revealed that sleep duration, depression, panic attacks, eating disorder, OCD, ADHD, words insulting, rate mental health, cyberbullying, dyslexia, bipolar disorder, and autism spectrum were significantly (*p* < 0.05) associated with anxiety in the studied population. Participants who are suffering from depression, panic attacks, eating disorder, OCD, ADHD, words insulting, dyslexia, bipolar disorder, and autism spectrum have a higher risk of anxiety at 9.422 (95% CI: 6.822–13.030), 10.403 (95% CI: 7.515–14.436), 4.542 (95% CI: 3.486–5.896), 4.361 (95% CI: 2.735–7.836), 2.168 (95% CI: 1.225–3.778), 1.284 (95% CI: 1.006–1.629), 0.970 (95% CI: 0.396–2.357), 0.343 (95% CI: 0.141–0.837), and 1.677 (95% CI: 0.674–4.238) respectively. Bipolar disorder and words insulting you were significant variables for the study participants (<0.05).

**Table 3:** Results of the final model for the association of anxiety and mental health disorders using a multivariable logistic regression model.

Item	Level	OR	95% CI	*p* value
Intercept	---	0.021	0.012–0.037	<0.0001
Sleep duration	0 day	Ref	Ref	<0.0001
	1 day	0.708	0.479–1.036	
	2 days	0.867	0.626–1.202	
	3 days	0.846	0.609–1.176	
	4 days	0.688	0.460–1.020	
	5 days	1.022	0.677–1.529	
	6 days	0.823	0.424–1.509	
	7 days	0.882	0.570–1.347	
Cyberbullying	Yes	Ref	Ref	<0.0001
	No	1.0194	0.706–1.495	
Dyslexia	No	Ref	Ref	<0.0001
	Yes	0.970	0.396–2.357	
Bi.Polar.Disorder	No	Ref	Ref	0.00402
	Yes	0.343	0.141–0.837	
Autism.spectrum	No	Ref	Ref	<0.0001
	Yes	1.677	0.674–4.238	
Depression	No	Ref	Ref	<0.0001
	Yes	9.422	6.822–13.030	
Panic attacks	No	Ref	Ref	<0.0001
	Yes	10.403	7.515–14.436	
Eating disorder	No	Ref	Ref	<0.0001
	Yes	4.542	3.486–5.896	
Obsessive compulsive disorder (OCD)	No	Ref	Ref	<0.0001
	Yes	4.361	2.735–7.836	
ADHD	No	Ref	Ref	<0.0001
	Yes	2.168	1.225–3.778	
Words insulting you	No	Ref	Ref	0.0352
	Yes	1.284	1.006–1.629	
Rate of mental health	Excellent	Ref	Ref	<0.0001
	Poor	5.868	3.634–9.631	
	Fair	4.525	3.024–6.963	
	Good	2.569	1.740–3.908	
	Very good	1.628	1.051–2.573	

### 3.5 Association of anxiety with behavior disorders

The results of the final model for the association of anxiety with explanatory variables of behavior disorders using a multivariable logistic regression model are listed in Table 4. The final multivariable model revealed that use of illegal drugs, prescribed medication, and internet use were significantly (*p* < 0.05) associated with anxiety in the studied population. Participants who use illegal drugs have a higher risk of anxiety at 2.623 (95% CI: 1.120–6.172) compared to those who did not use them. Participants who use prescribed medication have a higher risk of anxiety at 4.129 (95% CI: 1.991–8.564) compared to those who do not use it. Participants who use the internet have a higher risk of anxiety at 1.733 (95% CI: 1.321–2.274) compared to those who do not use it.

**Table 4:** Results of unconditional association of anxiety with the mental health disorders using an univariable logistic regression model.

Item	Level	OR	95% CI	*p* value
Intercept	---	0.096	0.088–0.104	<0.0001
Illegal drugs	No	Ref	Ref	0.00137
	Yes	2.623	1.120–6.172	
Prescribed medication	No	Ref	Ref	<0.0001
	Yes	4.129	1.991–8.564	
Internet	No	Ref	Ref	0.00016
	Yes	1.733	1.321–2.274	

## 4 Discussion

The findings of this study offer significant insights regarding the influence of socio-demographic and health-related factors on anxiety levels among university students in the UAE in the post-COVID-19 period. The core theoretical constructs of this study, cultural stressors and stigma, were not measured directly using dedicated scales. Instead, their influence was inferred based on the quantitative patterns observed in the data and contextualized within the established literature on Emirati society and mental health. Specifically, the persistent correlation between anxiety symptoms and variables such as family expectations about academic performance, fear of community judgment, or pressure to maintain a positive public image, was interpreted as a potential indicator of underlying cultural stressors. Similarly, the negative association between anxiety levels and help-seeking behavior, willingness to discuss mental health with peers, was interpreted as a potential manifestation of stigma.

Male students reported lower anxiety levels; however, this may reflect underreporting attributable to cultural norms that discourage emotional expression in men. Conversely, female students displayed higher levels of anxiety, likely stemming from biological, psychological, and societal pressures, which aligns with previous research conducted in similar conservative contexts (Abdulla et al., 2022; Alteneiji, 2023; Webb et al., 2024). The significant gender disparity in anxiety scores aligns with regional studies conducted in Saudi Arabia and Kuwait, which also report higher internalizing disorders among young women (Al-Garni et al., 2025; Alotaibi et al., 2024; Haggag et al., 2022; Mirza et al., 2021). This pattern is often interpreted through the lens of culturally prescribed gender roles. In the GCC region, young women may face unique pressures related to family honor (*ird*) and social reputation, which can internalize distress (Al-Darmaki, 2015; Al-Darmaki et al., 2016). Furthermore, while emotional expression might be more permitted for women, the pathways for seeking formal help for mental health issues may be more restricted due to stigma, potentially exacerbating anxious symptoms (Al-Krenawi and Graham, 2000; Fakhr el-Islam, 2008). Conversely, the lower reported anxiety among males may reflect cultural norms discouraging the expression of vulnerability, leading to under-reporting rather than a true absence of symptoms. Despite a noted increase in mental health awareness on a national scale, the stigma surrounding psychological distress continues to pose a significant barrier to help-seeking behaviors and early intervention.

Anxiety severity was significantly associated with age, income, disability status, and physical health conditions. Younger students, particularly those under 34, and those with lower household incomes (below AED 10,000), were more vulnerable to anxiety, likely due to fewer coping resources, limited financial stability, and life experience (Deasy et al., 2014; Rahman et al., 2020). Furthermore, students with disabilities or chronic health conditions reported markedly higher levels of anxiety, potentially due to the compounded challenges they face academically and socially. These findings underscore the necessity for inclusive support services that are specifically tailored to address the diverse needs of university populations.

Coping mechanisms have emerged as a crucial factor in understanding student anxiety. Numerous students have opted for social support and optimistic strategies instead of exhibiting feelings of helplessness. Nevertheless, the selection of coping styles is influenced by personal beliefs, such as an external locus of control or a belief in luck. Additionally, mental health comorbidities—including depression, panic attacks, obsessive-compulsive disorder (OCD), attention-deficit/hyperactivity disorder (ADHD), eating disorders, and autism spectrum disorder—are significantly correlated with heightened levels of anxiety severity. Furthermore, students who have encountered cyberbullying and those experiencing substantial daytime sleepiness also report elevated anxiety symptoms. These findings underscore the intricate relationship between comorbid conditions and anxiety, highlighting the essential need for thorough screening and the establishment of effective support systems (Jin et al., 2022; Mojtabai et al., 2015).

The study also investigated the relationship between behavioral disorders and anxiety. It identified risk behaviors, including excessive internet use, gaming, alcohol consumption, and smoking, that were associated with heightened anxiety severity; however, not all identified associations achieved statistical significance. These behaviors may serve as maladaptive coping strategies for students facing academic and social pressures (Ingledew et al., 1996; Rahman et al., 2020; Straup et al., 2022). Excessive internet use and prescribed medications were significantly linked to higher anxiety levels. The findings underscore the necessity for mental health interventions that focus on lifestyle changes, and they highlight the importance of early education regarding healthy coping mechanisms, particularly for adolescents and emerging adults within university environments (Fazel et al., 2014; Gronholm et al., 2023; Mills et al., 2020).

Although only a small percentage of participants reported a formal diagnosis of anxiety, approximately 8% exhibited severe anxiety symptoms as indicated by the Generalized Anxiety Disorder 7-item (GAD-7) scale. This discrepancy suggests a potential underdiagnosis of anxiety disorders and underscores the necessity for proactive screening within academic settings. The utilization of the GAD-7 provided a reliable and efficient means for identifying students at risk. Furthermore, logistic regression analyses elucidated the relationship between demographic and health-related variables and the severity of anxiety symptoms. These findings underscore the efficacy of the GAD-7 as a valuable mental health screening instrument in higher education (Plummer et al., 2016; Spitzer et al., 2006).

The study, while contributing valuable insights, is not without its limitations. The cross-sectional design of the research limits the ability to conclude causal relationships. Additionally, the reliance on self-reported data may introduce response bias, particularly in cultural contexts where mental health stigma remains prevalent. Although the GAD-7 is both validated and widely employed, it should not be viewed as a substitute for clinical diagnosis. Furthermore, the study sample predominantly comprised students in good health, which may affect the generalizability of the findings. Nevertheless, this research offers significant evidence regarding the predictors and patterns of anxiety among Emirati university students and emphasizes the necessity of culturally informed mental health support within higher education environments. Most notably, the constructs of cultural stress and stigma were inferred from behavioral correlations rather than measured directly through validated scales. While this inference is grounded in theoretical frameworks and the cultural context, it prevents definitive causal claims about their role. Future research should seek to develop and employ culturally validated instruments to directly quantify the levels and types of cultural stress and stigma experienced by Emirati youth to provide a more precise understanding of these mechanisms.

## 5 Conclusion

The findings of this study highlight the importance of culturally responsive, multi-faceted interventions to address anxiety among university students in the UAE. Effective strategies should include regular screening for anxiety and related health behaviors, increased visibility and accessibility of university counseling services, and the implementation of peer mentoring programs that align with local cultural values. Tailoring mental health support to the UAE context, such as incorporating Arabic-language resources, engaging families and religious leaders, and promoting group-based support systems, can reduce stigma and encourage help-seeking behavior. Additionally, skill-building initiatives that equip students with long-term coping strategies and emotional resilience are essential. Future research should complement these findings with qualitative studies to explore students’ lived experiences more deeply and inform the design of inclusive, sustainable mental health programs in higher education.

## Data Availability

The original contributions presented in the study are included in the article/supplementary material, further inquiries can be directed to the corresponding author.
